# Counterion‐Controlled Photocatalytic Doping of Organic Semiconductors

**DOI:** 10.1002/adma.72947

**Published:** 2026-04-02

**Authors:** Tiefeng Liu, Zesheng Liu, Ihor Sahalianov, Qiao He, Sang Young Jeong, Huotian Zhang, Qifan Li, Chi‐Yuan Yang, Junpeng Ji, Lize Bynens, Wouter Maes, Feng Gao, Han Young Woo, Martin Heeney, Glib Baryshnikov, Mats Fahlman, Simone Fabiano

**Affiliations:** ^1^ Laboratory of Organic Electronics Department of Science and Technology Linköping University Norrköping Sweden; ^2^ Wallenberg Initiative Materials Science for Sustainability Department of Science and Technology Linköping University Norrköping Sweden; ^3^ College of Education Sciences The Hong Kong University of Science and Technology (Guangzhou) Guangzhou China; ^4^ Department of Chemistry College of Science Korea University Seoul Republic of Korea; ^5^ Electronic and Photonic Materials Department of Physics, Chemistry, and Biology Linköping University Linköping Sweden; ^6^ Institute for Materials Research (IUMAT) Hasselt University Hasselt Belgium; ^7^ imec IUMAT diepenbeek Belgium; ^8^ Physical Sciences and Engineering Division King Abdullah University of Science and Technology (KAUST) Thuwal Kingdom of Saudi Arabia

**Keywords:** acridinium, aggregation, counterions, organic semiconductors, photocatalytic doping

## Abstract

Photocatalytic doping is a versatile and potentially sustainable strategy to control charge accumulation and transport in organic semiconductors (OSCs). In this process, light‐activated photocatalysts (PCs) act as electron shuttles, oxidizing or reducing OSCs under mild conditions, while redox‐inert salts supply counterions to stabilize the resulting charges. Although the energetics of PC/OSC systems are well studied, the influence of counterions has not yet been systematically examined. Here, we show that counterion size and interaction with the PC critically govern photocatalytic doping efficiency. Using acridinium‐based PCs with lithium salts of varying anion size, we find that smaller anions such as bis(fluorosulfonyl)imide (FSI^−^) suppress PC aggregation, enhance electron transfer, and yield conductivities up to 2000 S cm^−1^ in PBTTT derivatives. Spectroscopic and density functional theory (DFT) analyses show that FSI^−^ disrupts Acr‐Me^+^ stacking and increases its electron affinity by ∼0.1 eV relative to bulkier anions. These results uncover counterion size as a key design parameter for optimizing photocatalytic doping in OSCs.

## Graphical abstract

Counterion size is shown to actively control photocatalytic doping in organic semiconductors. Small anions suppress aggregation of acridinium photocatalysts, enhance electron transfer, and dramatically increase doping efficiency. This simple strategy enables conductivities up to 2000 S cm^−^^1^ in benchmark polymers, revealing counterions as a powerful yet overlooked design parameter for photocatalytic doping.

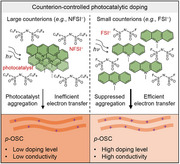

## 1 Introduction

Organic semiconductors (OSCs) are a versatile class of materials for applications ranging from light‐emitting diodes [1] and solar cells [2, 3] to thermoelectric [4, 5], transistors [6, 7], and bioelectronic devices [8, 9]. Their unique combination of mechanical flexibility, solution processability, chemical tunability, and compatibility with low‐temperature processing makes them valuable alternatives to traditional inorganic semiconductors [10, 11, 12, 13]. A key enabler of high performance in these applications is chemical doping, which modulates the electrical conductivity of OSCs by orders of magnitude and is essential for optimizing charge injection, extraction, and transport properties [14, 15].

Unlike doping in inorganic semiconductors, which typically involves substitutional impurities, doping in OSCs relies on electron transfer between the semiconductor and molecular dopants. Efficient doping requires precise alignment of frontier molecular orbitals, with high‐electron‐affinity (EA) dopants for *p*‐type doping and low‐ionization‐energy (IE) dopants for *n*‐type doping [16, 17, 18]. However, these energetic requirements often come at the cost of chemical reactivity and instability, motivating the development of milder strategies such as adduct‐based doping [3], proton‐coupled electron transfer doping [19], coupled‐reaction doping [20], and light‐induced doping [21, 22]. Yet, many of these approaches still depend on dopants that are difficult to process, moisture‐sensitive, or require activation by heat [23, 24, 25], radiation [26], or metal/organometallic catalysts [27, 28, 29].

**FIGURE 1:**
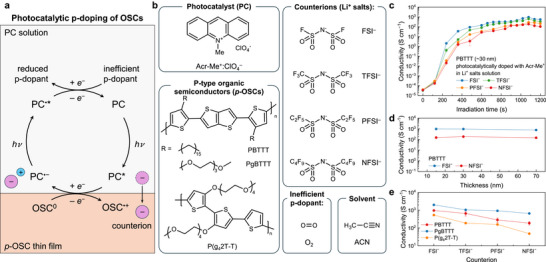
Photocatalytic doping mechanism and counterion‐dependent conductivity in *p*‐OSCs. (a) Schematic of the photocatalytic doping process, where excited PCs oxidize the *p*‐OSC and are regenerated by inefficient dopants. (b) Chemical structures of Acr‐Me^+^:ClO_4_^−^ (PC), PBTTT, PgBTTT, and P(g_4_2T‐T) (*p*‐OSCs), counterions, and dopant/solvent used in this work. (c) Conductivity of a ∼30 nm PBTTT film photocatalytically doped by Acr‐Me^+^ in the presence of various counterions under 455 nm LED irradiation (50 mW cm^−2^). (d) Conductivity of PBTTT films with varying thickness doped by Acr‐Me^+^ in the presence of FSI^−^ or NFSI^−^. (e) Maximum electrical conductivity of photocatalytically doped PBTTT, PgBTTT, and P(g_4_2T‐T) as a function of counterion.

Recently, we introduced photocatalytic doping as a versatile and potentially sustainable strategy to control charge accumulation and transport in OSCs. Unlike conventional doping approaches that rely on highly reactive dopants, our method employs light‐activated photocatalysts (PCs) as electron shuttles, enabling both oxidation and reduction of OSCs under mild conditions with widely available weak/inefficient dopants such as oxygen (for *p*‐doping) or simple amines (for *n*‐doping) [30]. In this process, solution‐phase PCs, such as acridinium derivatives, remain electrochemically inactive in the dark but become strong oxidants or reductants upon photoexcitation, transferring electrons to or from the OSC (Figure 1a). Their regeneration is achieved by weak sacrificial dopants, while redox‐inert salts such as lithium bis(trifluoromethanesulfonyl)imide (LiTFSI) provide counterions that stabilize polarons on the polymer chains, thereby maintaining charge neutrality without disrupting polymer packing. Notably, the PC is recycled during the cycle, with only the weak/inefficient dopant and salt being consumed. The efficiency of the photocatalytic doping process depends on the energetics of the PC/OSC system, as the PC affects only the kinetics of electron transfer, while the salt has so far been regarded as a passive counterion without an active role.

Here, we show that the additional salt not only compensates charges in the doped OSCs but also interacts electrostatically with the ionic PCs in solution, thereby influencing the overall doping efficiency. This behavior contrasts with conventional molecular doping strategies, in which counterions are typically introduced after electron transfer and are mainly discussed in terms of their impact on solid‐state properties, such as film microstructure [31, 32, 33] or energetic disorder [34], rather than as active participants in the charge‐generation step itself. Specifically, we found that small counterions such as bis(fluorosulfonyl)imide (FSI^−^) disrupt the aggregation of 10‐methylacridinium (Acr‐Me^+^), as revealed by density functional theory (DFT) simulations and optical measurements. This suppression of aggregation yields *p*‐doped OSCs with higher charge density and conductivity than those obtained with larger counterions such as bis(nonafluorobutanesulfonyl)imide (NFSI^−^), as confirmed by absorption spectroscopy, X‐ray photoelectron spectroscopy (XPS), and electron paramagnetic resonance (EPR). These results provide a simple strategy to tune the doping ability of PCs and reveal an unexpected active role for counterions in the photocatalytic doping of OSCs.

## 2 Results and Discussion

To study the effect of counterions on photocatalytic doping, we focused on the oxidation of poly(2,5‐bis(3‐hexadecylthiophen‐2‐yl)thieno[3,2‐*b*]thiophene) (PBTTT) using Acr‐Me^+^ as the PC, chosen for its commercial availability, air stability, and strong oxidizing capabilities in the excited state [35]. Four Li^+^ salts with systematically increasing anion size, namely FSI^−^, TFSI^−^, PFSI^−^ (bis(pentafluoroethanesulfonyl)imide), and NFSI^−^, were selected to probe size‐dependent effects. The chemical structure of Acr‐Me^+^, PBTTT, and the Li^+^ salts are shown in Figure 1b. Briefly, upon illumination at 455 nm, Acr‐Me^+^ is promoted to its excited state (Acr‐Me^+*^), oxidizes PBTTT via reductive quenching, and is subsequently regenerated (reoxidized) by molecular oxygen from ambient air. The conductivity of PBTTT increases progressively with irradiation time until it saturates (Figure 1c; Table S1). Consistent with our previous reports [30], photocatalytic *p*‐doping of PBTTT with Acr‐Me^+^ in the presence of TFSI^−^ yields conductivities of about 659 ± 188 S cm^−1^, over two order of magnitude higher than in the absence of Li^+^ salts (1.31 ± 0.23 S cm^−1^, see Figure S1), highlighting the critical role of counterions in enabling efficient doping [36, 37]. Strikingly, the conductivity strongly depends on the size of the counterion: larger anions such as NFSI^−^ reduced the conductivity to ∼187 ± 40 S cm^−1^, whereas smaller anions such as FSI^−^ markedly enhanced it, reaching 970 ± 107 S cm^−1^ (Figure 1c), among the highest values reported for this benchmark polymer [31, 32, 38]. Importantly, PBTTT films doped with FSI^−^ show comparable stability to those doped with larger counterions under nitrogen and retain their conductivity more effectively under ambient conditions (Figure S2). Notably, the same ion‐size‐dependent conductivity trend is observed in both thin and thick films (Figure 1d; Figure S3), indicating that the observed differences are not governed by ion penetration depth or mass‐transport limitations. This trend is general and is observed for other acridinium‐based PCs (Figures S4 and S5), as well as for the glycolated derivative of PBTTT (namely, PgBTTT), where incorporation of FSI^−^ yields an even higher conductivity of 2005 ± 160 S cm^−1^ (Figure S6), likely enabled by its lower IE compared to PBTTT (4.41 vs. 4.95 eV, Figure S7) and the reduced defect density associated with its homocoupling‐free synthesis [39]. This pronounced ion‐size dependence stands in sharp contrast to other doping strategies, such as ionic‐exchange doping [36] and proton‐coupled doping [19], where larger counterions have typically been associated with higher conductivities, pointing instead to a fundamentally different doping mechanism.

**FIGURE 2:**
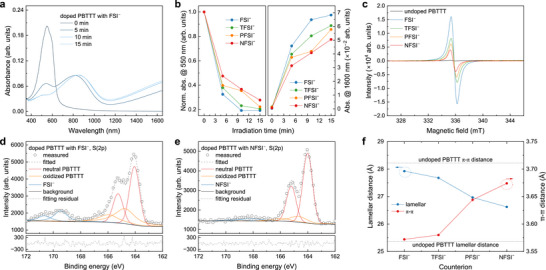
Spectroscopic and structural characterization of photocatalytically doped PBTTT. (a) Absorption spectra of PBTTT films photocatalytically doped by Acr‐Me^+^ with FSI^−^. (b) Neutral (550 nm) and polaron (1600 nm) absorption evolution under illumination for different counterions. (c) EPR spectra of undoped and doped PBTTT films using Acr‐Me^+^ in the presence of various Li^+^ salts. (d,e) XPS S(2p) spectra of PBTTT doped in the presence of FSI^−^ (d) and NFSI^−^ (e). (f) *π*–*π* stacking and lamellar spacing of doped PBTTT with different counterions; dashed lines mark undoped values.

To understand how counterion size affects the electrical conductivity and doping efficiency of PBTTT photocatalytically doped with Acr‐Me^+^, we measured the absorption spectra of the doped films (Figure 2a; Figure S8). With the smallest counterion (FSI^−^), the characteristic absorption peak of pristine PBTTT at 550 nm rapidly bleaches, while the polaron absorption in the infrared region increases strongly within the first 5 min of illumination. After 10 min, the neutral absorption peak was almost completely suppressed, indicating complete doping. In contrast, with the largest counterion (NFSI^−^), bleaching of the neutral absorption peak proceeds much more slowly, and even after 15 min, a residual peak remained, suggesting less effective doping. These absorption trends align with the conductivity data presented in Figure 1c, where films doped with FSI^−^ show the fastest conductivity increase within the first 5 min of illumination. The temporal evolution of the neutral absorption peak at 550 nm and the polaron peak at 1600 nm under continuous illumination is shown in Figure 2b. Polaron formation in the photocatalytically doped PBTTT films is further supported by EPR measurements (Figure 2c), where the EPR signal intensity systematically decreases as the counterion size increases from FSI^−^ to NFSI^−^. No EPR signal was detected in pristine PBTTT, confirming the absence of polarons in the undoped state.

To assess how counterion size affects doping efficiency, we performed XPS on PBTTT films photocatalytically doped with Acr‐Me^+^. In the undoped state, the sulfur S(2p) signal of PBTTT appears as a symmetric spin‐split doublet at 164.0 and 165.2 eV, corresponding to the sulfur atoms in the thiophene backbone (Figure S9a). Upon doping, the S(2p) peaks become asymmetric, exhibiting tailing toward higher binding energies (Figure 2d,e; Figure S9b,c), consistent with the formation of delocalized positive charges along the polymer backbone and modifications to the local electronic environment [40, 41]. Additionally, two new peaks emerged between 167–171 eV, attributed to sulfur atoms from the counterions. By fitting the PBTTT S(2p) region with two spin‐split doublets corresponding to neutral and oxidized polythiophene, we quantified the doping level as the fraction of oxidized PBTTT relative to the total polymer signal [42]. This analysis yields values of 41%, 37%, 33%, and 16% for PBTTT doped with FSI^−^, TFSI^−^, PFSI^−^, and NFSI^−^, respectively (see Methods for details). This trend is corroborated by the oxygen O(1s), nitrogen N(1s), and fluorine F(1s) signals (Figure S10), whose relative intensities follow the same order, confirming the progressive reduction in counterion incorporation with increasing anion size. Seebeck coefficient measurements further support these findings (Figure S11). Films doped with FSI^−^ exhibit the lowest Seebeck coefficient (23.44 ± 0.80 µV K^−1^), while those with NFSI^−^ show the highest (38.62 ± 0.45 µV K^−1^). This is consistent with the trends observed by XPS, EPR, and absorption spectroscopy, reinforcing the conclusion that smaller counterions enable more efficient photocatalytically doping with Acr‐Me^+^.

We next investigated the structural changes in PBTTT thin films induced by photocatalytic doping using grazing‐incidence wide‐angle X‐ray scattering (GIWAXS). Undoped PBTTT films exhibit a predominant edge‐on orientation, with the polymer backbone oriented nearly perpendicular to the substrate. Doping does not alter this molecular orientation, but it induces significant structural changes. Specifically, we observed a notable expansion of the lamellar (100) spacing and a slight contraction of the *π*–*π* (010) stacking distance, consistent with counterion insertion primarily within the side‐chain regions [43]. As the counterion size increases from FSI^−^ to NFSI^−^, the *π*–*π* stacking distance increases from 3.57 to 3.67 Å, while the lamellar spacing decreases from 27.92 to 26.62 Å (Figure 2f). These structural changes correlate with the observed reduction in doping level. Full 2D GIWAXS patterns and peak fitting analyses are provided in Figures S12–S16 and Table S2.

**FIGURE 3:**
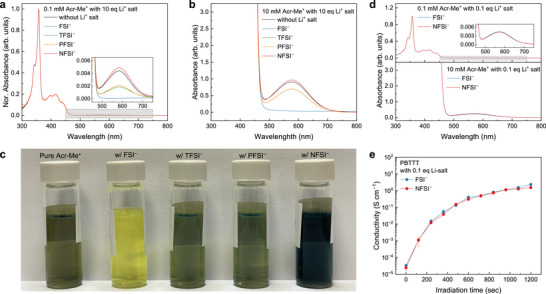
Effect of counterions on the aggregation and photocatalytic activity of Acr‐Me^+^. (a,b) Absorption spectra of diluted (0.1 mm, a) and concentrated (10 mm, b) Acr‐Me^+^ solutions mixed with 10 equivalents of various counterions; inset in (a) shows the 450–750 nm region. (c) Photographs of 10 mm Acr‐Me^+^ solutions mixed with different counterions. (d) Absorption spectra of 0.1 and 10 mM Acr‐Me^+^ solutions mixed with 0.1 eq FSI^−^ or NFSI^−^. (e) Conductivity of PBTTT films photocatalytically doped by Acr‐Me^+^ in the presence of 0.1 eq FSI^−^ or NFSI^−^ under 455 nm LED irradiation (50 mW cm^−2^).

To understand the origin of the ion‐size dependence in photocatalytic doping efficiency of Acr‐Me^+^, we investigate its interaction with different counterions. As shown in Figure 3a, Acr‐Me^+^ in dilute solution (0.1 mm) exhibits two main absorption peaks at 358 and 416 nm, in agreement with reported spectra of acridinium ions [44, 45]. Upon mixing Acr‐Me^+^ with the various Li^+^ salts, the absorption features in the 300–470 nm range remain largely unchanged, indicating that the anions do not significantly perturb the ground‐state electronic structure of the PC. However, we observed a gradual reduction in the broad absorption band between 500–700 nm as the counterion size decreased from NFSI^−^ to FSI^−^. This effect becomes more pronounced in concentrated solutions (10 mm), as shown in Figure 3b, and is visually evident in the corresponding solution colors (Figure 3c): Acr‐Me^+^ appears bright yellow when mixed with FSI^−^ and dark green with NFSI^−^. Note that all Li^+^ salts are transparent in the 300–800 nm range (Figure S17) and are redox‐inert, as they alone induce negligible changes in both electrical conductivity and absorption of PBTTT films (Figures S18 and S19).

This 500–700 nm band is attributed to charge‐transfer absorption within aggregated acridinium species [46, 47], suggesting that smaller counterions more effectively suppress Acr‐Me^+^ aggregation. This interpretation is further supported by fluorescence measurements (Figure S20), where mixtures with larger anions exhibit blue‐shifted emission, consistent with the formation of acridinium aggregates [48]. Time‐resolved fluorescence also shows that Acr‐Me^+^ exhibits longer excited‐state lifetimes in the presence of smaller anions: 31.28 ns for FSI^−^, 26.99 ns for TFSI^−^, 24.08 ns for PFSI^−^, and 21.72 ns for NFSI^−^ (Figure S21), which is beneficial for photocatalytic doping. Notably, we also found that this aggregation behavior influences the EA of Acr‐Me^+^, as shown in Figure S22. Pristine (aggregated) Acr‐Me^+^ molecules exhibit an EA of ‐4.35 eV, which remains unchanged in the presence of NFSI^−^ (‐4.37 eV). In contrast, the use of FSI^−^ increases the EA to ‐4.51 eV, consistent with the behavior of monomeric (non‐aggregated) species and in line with the computed trend (see below). Although modest, this shift in EA may enhance electron transfer—consistent with our previous observations that ∼0.1–0.2 eV differences in the EA of different PCs can yield several‐fold increases in conductivity [30]—and, together with the higher concentration of available PC molecules, likely account for the improved doping efficiency observed with smaller counterions. Finally, when Acr‐Me^+^ was mixed with 0.1 eq of LiFSI or LiNFSI, similar aggregation behavior was observed, as indicated by comparable absorbance in the 500–700 nm range (Figure 3d; absorbance spectra for different Li^+^ salt equivalents are shown in Figure S23), which resulted in similar conductivity values in the corresponding doped films (Figure 3e). Together, these findings highlight the critical influence of counterion size on Acr‐Me^+^ aggregation, and consequently, on the efficiency of the photocatalytic doping process.

**FIGURE 4:**
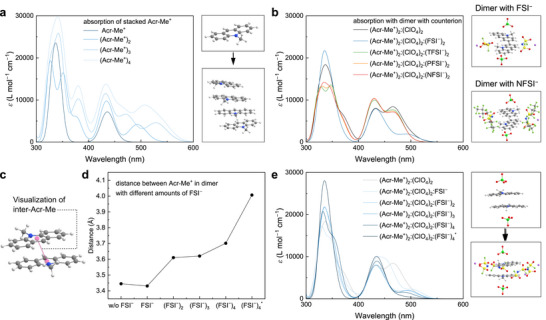
DFT analysis of Acr‐Me^+^ aggregation and ion‐molecule interactions. (a) Optimized geometries and simulated absorption spectra of Acr‐Me^+^ stacks (one to four molecules). (b) Simulated absorption spectra of Acr‐Me^+^ dimers with ClO_4_^−^ or various counterions (FSI^−^, TFSI^−^, PFSI^−^, NFSI^−^). (c) Intermolecular distance between Acr‐Me^+^ units in the same dimers. (d) Acr‐Me^+^–Acr‐Me^+^ distance as a function of FSI^−^ concentration. (e) Simulated absorption spectra of dimers surrounded by increasing amounts of FSI^−^.

To gain further insight into this phenomenon, we performed DFT calculations to examine how different counterions influence the absorption spectra of both individual and aggregated Acr‐Me^+^ molecules (Figures S24, S25 and Note S1). The simulations indicate that the experimentally observed absorption band between 450 and 700 nm originates primarily from Acr‐Me^+^ aggregation (Figure 4a; Figure S24), in agreement with previous reports [46, 47]. Among the counterions studied, only FSI^−^ is found to disrupt this aggregation‐induced absorption feature (Figure 4b; Figures S25–S27). In contrast to bulkier counterions such as TFSI^−^, PFSI^−^, and NFSI^−^, which stabilized Acr‐Me^+^ stacking via non‐covalent van der Waals interactions, the smaller FSI^−^ ion perturbs the stacking geometry when positioned near or between Acr‐Me^+^ dimers (Figures S26 and S27). This disruption reduces intermolecular interactions (Figure 4c,d) and significantly attenuates the absorption band in the 470–500 nm range, a region particularly sensitive to stacking distance (Figure 4e; Figure S28). Simulations further show that increasing the number of FSI^−^ ions leads to greater structural distortion and further suppression of the aggregation signature. In contrast, larger counterions preserve the stacked configuration and its associated spectral features. DFT calculations reveal a ∼0.1 eV increase in the ground‐state EA of Acr‐Me^+^ in the presence of FSI^−^, relative to pristine Acr‐Me^+^ or Acr‐Me^+^ associated with bulkier NFSI^−^, consistent with reduced aggregation and in line with experimental observations (Figure S29 and Table S3).

## 3 Conclusion

In summary, we elucidated the critical role of counterions in the photocatalytic doping of OSCs. While traditionally regarded as passive charge compensators, counterions are shown here to directly interact with the ionic PC in solution, significantly modulating doping efficiency. Specifically, we demonstrated that smaller anions such as FSI^−^ effectively disrupt the aggregation of Acr‐Me^+^, leading to enhanced photocatalytic activity. This reduced aggregation correlates with longer excited‐state lifetimes, higher EA, and ultimately more efficient charge transfer to the OSC. As a result, *p*‐doped PBTTT and its glycolated derivatives exhibit markedly higher doping levels and electrical conductivity when FSI^−^ is used as the counterion, reaching values up to 2000 S cm^−1^, compared to when bulkier anions such as NFSI^−^ are employed (658 S cm^−1^). Notably, a similar counterion‐size dependence is observed across other Acr‐based PCs, suggesting that this effect is general. However, the microscopic origin of this trend may vary among derivatives, as factors such as PC aggregation behavior and excited‐state localization can differ between acridinium systems. Elucidating these mechanisms will require dedicated studies for each PC. Overall, these findings reveal the choice of counterion as an overlooked yet powerful design parameter for tuning photocatalytic doping and offer a straightforward, generalizable strategy for enhancing the performance of photocatalytically doped OSCs.

## 4 Methods

### 4.1 Materials and Sample Preparation

PBTTT (*M*_w_ = 74 kDa) was synthesized following previously reported procedures [49]. Homocoupling‐free PgBTTT was synthesized according to the literature [39], and its weight‐average molar mass, as determined by diffusion NMR using a PgBTTT‐based calibration curve, was 18 kDa. P(g_4_2T‐T) (*M*_w_ = 20 kDa) were purchased from 1‐Materials, while Acr‐Me^+^:ClO_4_^−^ and the Li^+^ salts with different anions were obtained from TCI. All materials were used as received. PBTTT was dissolved in 1,2‐dichlorobenzene (10 mg ml^−1^), whereas PgBTTT and P(g_4_2T‐T) were each dissolved in chloroform (10 mg ml^−1^). Thin films of the p‐type OSCs were fabricated by spin‐coating the corresponding solution onto the substrates, followed by thermal annealing at 180°C for 20 min in a N_2_‐filled glovebox. The films were then allowed to cool slowly to room temperature. The PC solutions were prepared by dissolving Acr‐Me^+^ and Li^+^ salts in acetonitrile. The typical concentrations of Acr‐Me^+^ and Li^+^ salts were 10 and 100 mm, respectively.

### 4.2 Photocatalytic Doping

The photocatalytic doping process was carried out as previously reported [30]. In a typical procedure, OSC thin films were immersed in the dopant solution and irradiated with 455 nm blue light (50 mW cm^−2^). A heat sink was placed beneath the films to minimize heating and reduce solvent evaporation. The process was performed in air, where molecular oxygen served as the weak p‐dopant. After irradiation, the films were rinsed with acetonitrile and dried under a N_2_ flow.

### 4.3 Electrical and Optical Characterization

Electrical conductivity was measured using a four‐probe setup with a channel width/length of 4000/200 µm. Seebeck coefficients were measured inside a N_2_‐filled glovebox using a pair of Peltier elements to generate a controlled temperature gradient. All electrical measurements were carried out using a Keithley 4200‐SCS semiconductor characterization system.

OSC thin films were prepared on glass substrates, and solution samples were placed in quartz cuvettes. Ultraviolet‐visible‐near infrared (UV—vis–NIR) absorbance spectra were recorded using a PerkinElmer Lambda 950 spectrophotometer with a wavelength step of 2 nm.

### 4.4 XPS and EPR Spectroscopy

PBTTT films were spin‐cast onto Au‐coated glass substrates for XPS analysis. Measurements were performed using a Scienta ESCA 200 system equipped with a SES 200 electron analyzer under ultrahigh‐vacuum conditions (1 × 10^−10^ mbar). A monochromatic Al Ka X‐ray source (1486.6 eV) was used. All spectra were collected at normal emission and calibrated against a sputter‐cleaned Au film, with the Au 4f peak set to 84.0 eV. The PBTTT S(2p) region was analyzed by fitting two spin‐orbit‐split doublets corresponding to neutral and oxidized PBTTT. The energy separation between the S(2p_3/2_) and S(2p_1/2_) components was fixed at 1.2 eV, with the S(2p_1/2_) peak constrained to have half the area and the same full width at half maximum as the corresponding S(2p_3/2_) peak. The oxidized PBTTT doublet was shifted by 0.7 eV relative to the neutral PBTTT doublet. The doping level was determined from the ratio of the integrated area of the oxidized PBTTT doublet to the total PBTTT S(2p) peak area. Importantly, varying the assumed energy shift of the oxidized PBTTT doublet by ∼14% (i.e., 0.7 ± 0.1 eV) results in only a ∼3%–4% change in the extracted doping level, confirming the robustness of the analysis.

For EPR measurements, PBTTT films were spin‐cast onto PET substrates, cut into 2 × 20 mm pieces, and sealed in N_2_‐filled quartz tubes. EPR spectra were recorded at room temperature under dark conditions using a SPINSCAN X spectrometer from Linev Systems. The modulation frequency, microwave power, and microwave frequency were set to 100 kHz, 1 mW, and 9.46 GHz, respectively. Spectra were acquired with a modulation width of 0.7 mT and a sweep time of 50 s.

### 4.5 Photoluminescence Spectroscopy

Photoluminescence spectra were recorded using a fluorescence spectrometer (FLS1000, Edinburgh Instruments) equipped with a 450 W xenon lamp and a PMT‑980 photomultiplier detector. Time‐resolved photoluminescence was measured using a 375 nm picosecond pulsed diode laser (HPL‐375).

### 4.6 Cyclic Voltammetry

Cyclic voltammetry (CV) measurements were performed using a BioLogic SP‐200 potentiostat. A 0.1 m solution of Bu_4_NPF_6_ in acetonitrile served as the electrolyte. OSC films spin‐cast on clean gold substrates were used as working electrodes, with a platinum mesh and a saturated Ag/AgCl electrode serving as counter and reference electrode, respectively. The potential was calibrated by a standard ferrocene sample, and all scans were recorded at a scan rate of 50 mV s^−1^.

### 4.7 GIWAXS Measurements

PBTTT films were spin‐cast onto silicon substrates, and measurements were carried out at the 9A U‐SAXS beamline of the Pohang Accelerator Laboratory (Republic of Korea) using a beam energy of 11.07 keV and an incident angle of 0.12°. All samples were measured under vacuum with an exposure time of 10 s.

### 4.8 DFT Calculations

All quantum‐chemical calculations were performed using the Gaussian 16 software package [50]. Geometry optimizations were carried out using the B3LYP functional with 10% Hartree‐Fock exchange, the 6–31G(d) basis set [51, 52], and Grimme's D3 dispersion correction [53]. An implicit acetonitrile solvent was modeled using the polarizable continuum model (PCM) [54]. Absorption spectra were simulated with time‐dependent DFT (TD‐DFT) [55] considering the first 50 electronic transitions, and S_1_ excited‐state geometries were optimized at the same level of theory. Visualization of molecular structures and orbitals was performed using Chemcraft software [56].

## Author Contributions

T.L. and S.F. conceived and designed the project. T.L. performed the optical measurements and recorded the EPR spectra. I.S. and G.B. carried out the DFT calculations and simulations. Z.L. and M.F. recorded and analyzed the XPS data. S.Y.J. and H.Y.W. collected the GIWAXS data, while T.L. and C.‐Y.Y. performed the data analysis. T.L. and J.J. performed the electrical measurements. Q.L. performed the CV measurements. H.Z. and F.G. conducted the photoluminescence experiments. Q.H. and M.H. synthesized PBTTT, while L.B. and W.M. synthesized PgBTTT. S.F. supervised the project. T.L. and S.F. wrote the manuscript. All authors discussed the results and contributed to the final version of the manuscript.

## Conflicts of Interest

The authors declare no conflicts of interest.

## Supporting information




**Supporting File**: adma72947‐sup‐0001‐SuppMat.pdf.

## Data Availability

The data that support the findings of this study are available from the corresponding author upon reasonable request.
