# Supplementary material for: Counterion‐Controlled Photocatalytic Doping of Organic Semiconductors
Source: Adv Mater. 2026 Apr 2;38(25):e72947. doi: 10.1002/adma.72947 (PMC13137766; doi:10.1002/adma.72947)
Supplement: Supplementary file 1 — Supporting File: adma72947‐sup‐0001‐SuppMat.pdf. [file ADMA-38-e72947-s001.pdf]

Counterion-Controlled Photocatalytic Doping of Organic Semiconductors

Tiefeng Liu[1,2], Zesheng Liu[1], Ihor Sahalianov[1,2], Qiao He[3], Sang Young Jeong[4], Huotian Zhang[5], Qifan Li[1], Chi-Yuan Yang[1], Junpeng Ji[1], Lize Bynens[6], Wouter Maes[6], Feng Gao[5], Han Young Woo[4], Martin Heeney[7], Glib Baryshnikov[1,2], Mats Fahlman[1], Simone Fabiano[1,2*]

[1]Laboratory of Organic Electronics, Department of Science and Technology, Linköping University, SE-60174 Norrköping, Sweden. E-mail: simone.fabiano@liu.se

[2]Wallenberg Initiative Materials Science for Sustainability, Department of Science and Technology, Linköping University, SE-60174 Norrköping, Sweden.

[3]College of Education Sciences, The Hong Kong University of Science and Technology (Guangzhou), 511453 Guangzhou, China.

[4]Department of Chemistry, College of Science, Korea University,136-713 Seoul, Republic of Korea.

[5]Electronic and Photonic Materials, Department of Physics, Chemistry, and Biology, Linköping University, Linköping SE-58183, Sweden.

[6]Hasselt University, Institute for Materials Research (imo-imomec), Martelarenlaan 42, B-3500, Hasselt, Belgium.

[7]Physical Sciences and Engineering Division, King Abdullah University of Science and Technology (KAUST), Thuwal 23955-6900, Kingdom of Saudi Arabia.

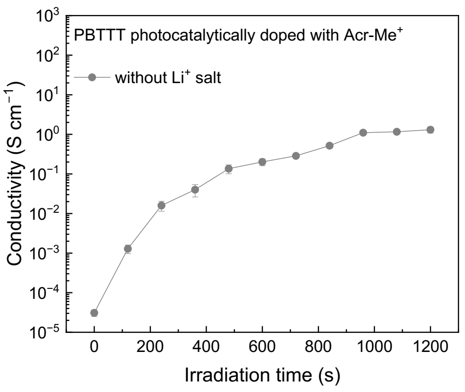

Figure S1. Electrical conductivity of a ~30 nm PBTTT film photocatalytically doped with Acr-Me$^+$ in the absence of Li$^+$ salts under 455 nm LED irradiation (50 mW cm^{-2}).

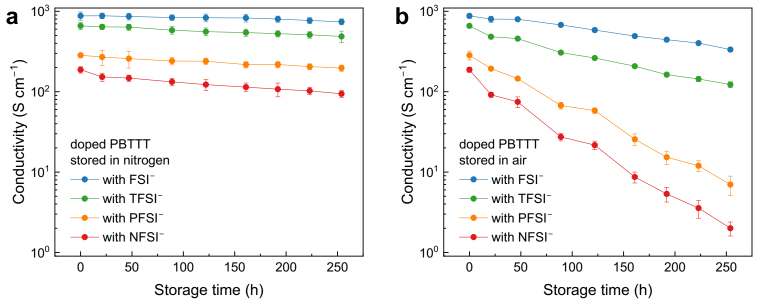

Figure S2. Stability of photocatalytically doped PBTTT with different counterions. Electrical conductivity evolution of doped PBTTT films stored under (a) nitrogen-filled glovebox and (b) ambient laboratory conditions (≈22 °C, ~40% RH).

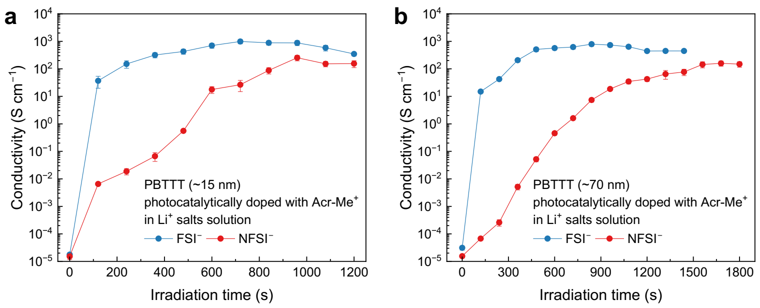

Figure S3. Electrical conductivity of PBTTT (a) thin (~15 nm) and (b) thick (~70 nm) films photocatalytically doped with Acr-Me$^+$ in the presence of FSI$^-$ or NFSI$^-$ under 455 nm LED irradiation (50 mW cm^{-2}).

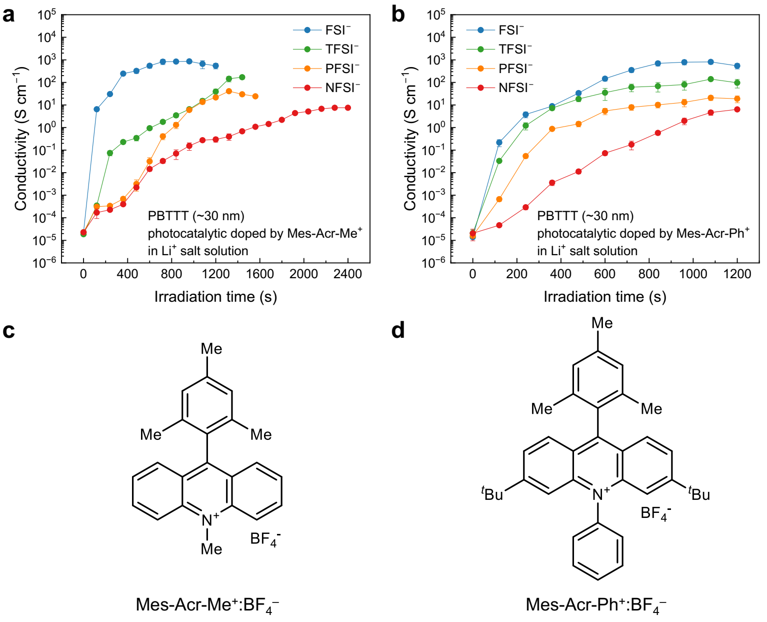

Figure S4. Electrical conductivity of PBTTT films (~ 30 nm) photocatalytically doped with (a) Mes-Acr-Me$^+$ and (b) Mes-Acr-Ph$^+$ in the presence of various counterions under 455 nm LED irradiation (50 mW cm^{-2}). The chemical structures of Mes-Acr-Me+ and Mes-Acr-Ph+ (both with BF4− as the counterion) are shown in (c) and (d).

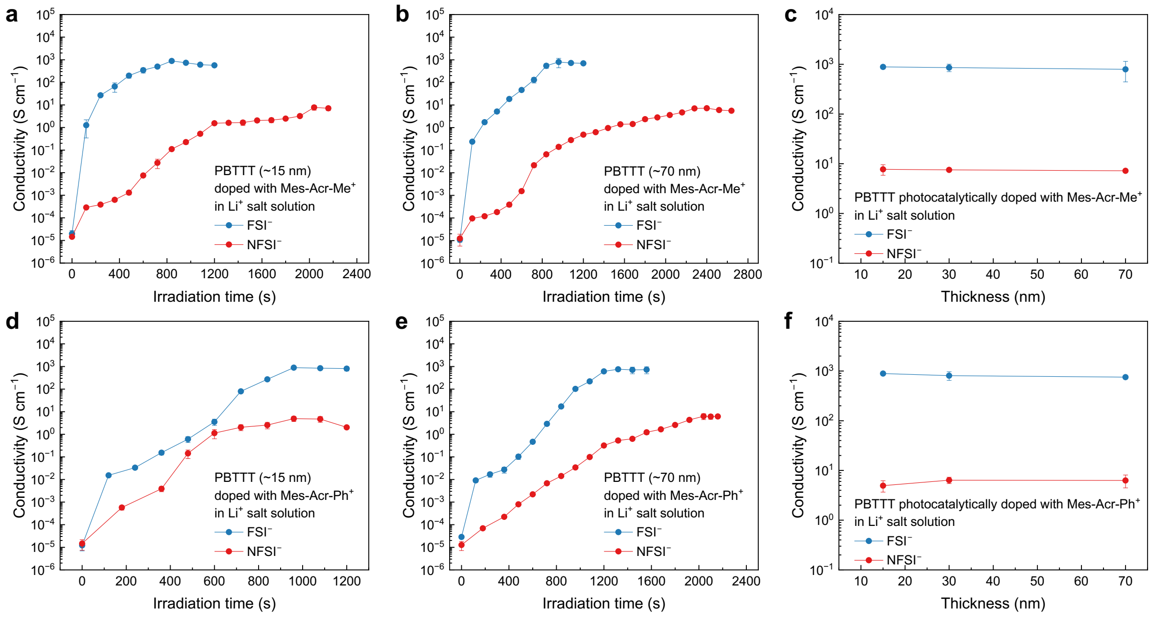

Figure. S5. (a) Thin (~15 nm) and (b) thick (~70 nm) PBTTT films photocatalytically doped with Mes-Acr-Me$^+$ in the presence of FSI$^-$ or NFSI$^-$. (c) Electrical conductivity of PBTTT films with varying thickness doped by Mes-Acr-Me$^+$ in the presence of FSI$^-$ or NFSI$^-$. (d) Thin (~15 nm) and (e) thick (~70 nm) PBTTT films photocatalytically doped with Mes-Acr-Ph$^+$ in the presence of FSI$^-$ or NFSI$^-$. (f) Electrical conductivity of PBTTT films with varying thickness doped by Mes-Acr-Ph$^+$ in the presence of FSI$^-$ or NFSI$^-$. Photocatalytic doping was performed under 455 nm LED irradiation (50 mW cm^{-2}).

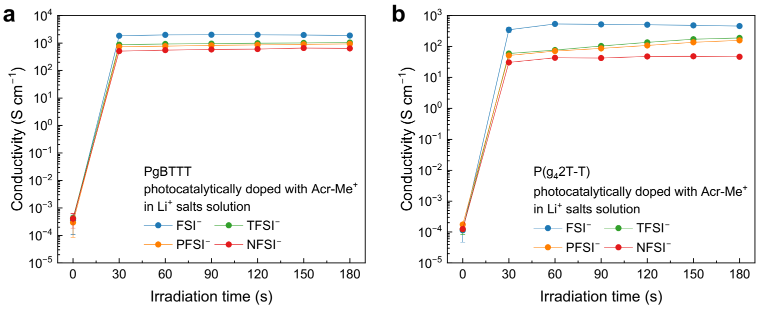

Figure S6. Electrical conductivity of (a) PgBTTT and (b) P($g_4$2T-T) films photocatalytically doped with Acr-Me^+ in the presence of various counterions under 455 nm LED irradiation (50 mW cm^{-2}).

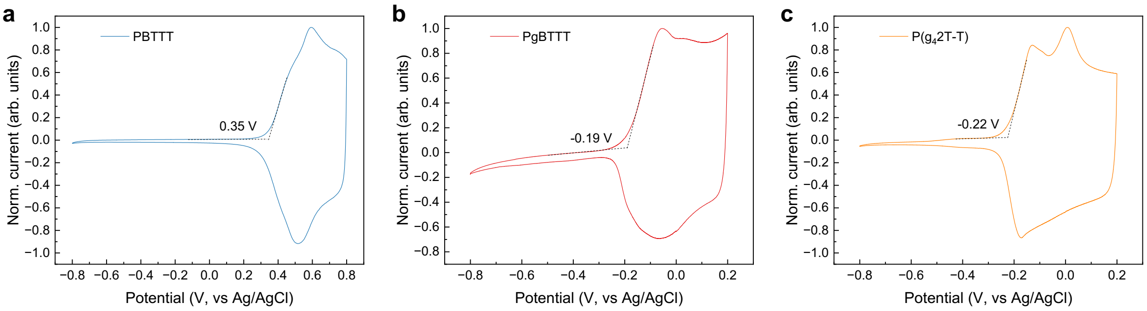

Figure S7. Cyclic voltammograms of (a) PBTTT, (b) PgBTTT, and (c) P($g_4$2T-T) films recorded in 0.1 M Bu_4NPF_6/acetonitrile solution at a scan rate of 50 mV s^{-1}.

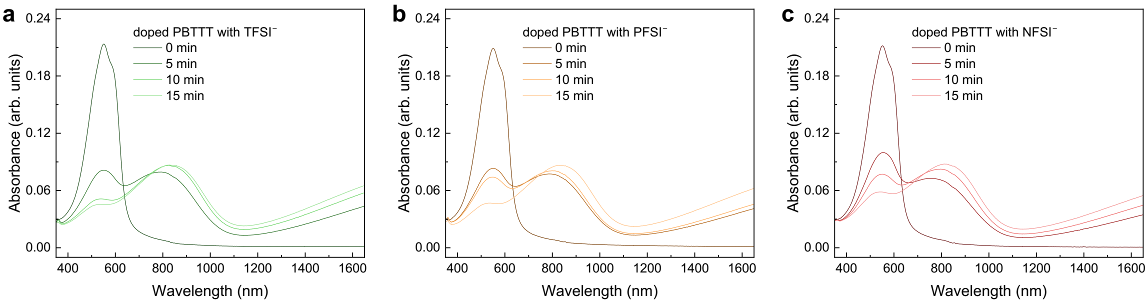

Figure S8. Absorption spectra of PBTTT films photocatalytically doped with Acr-Me^+ in the presence of (a) $TFSI^-$, (b) $PFSI^-$, and (c) $NFSI^-$.

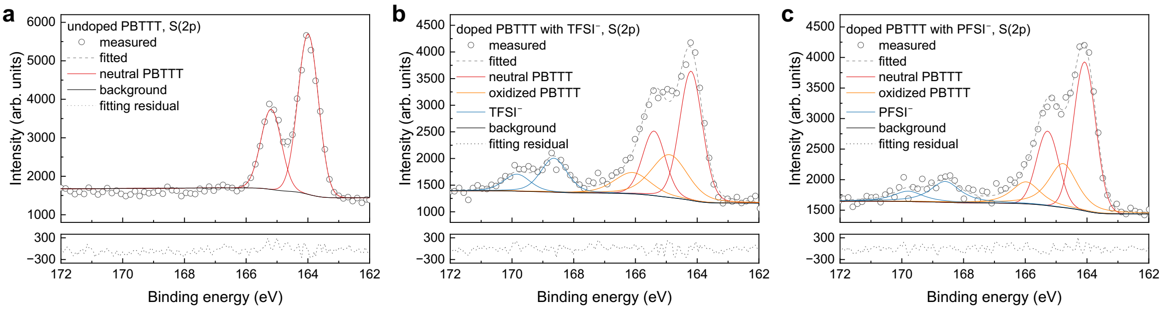

Figure S9. XPS S(2p) spectra of undoped (a) and photocatalytically doped PBTTT films in the presence of (b) $TFSI^{-}$ and (c) $PFSI^{-}$.

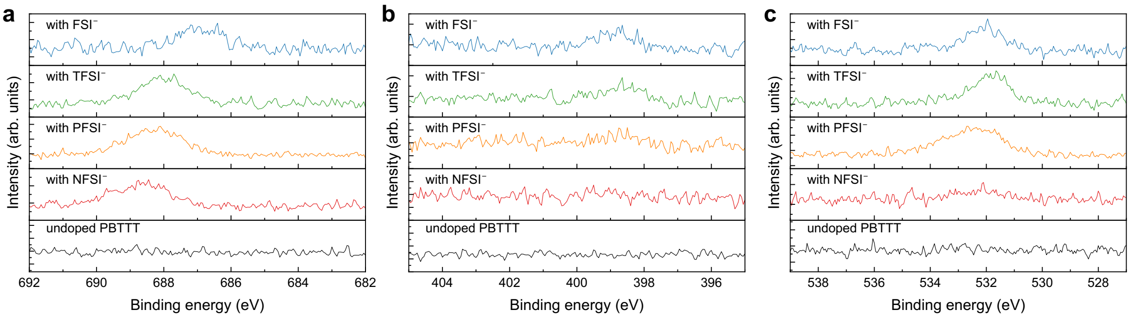

Figure S10. XPS (a) F(1s), (b) N(1s), and (c) O(1s) spectra of photocatalytically doped PBTTT films in the presence of different counterions. No corresponding peaks were detected in the undoped PBTTT films, confirming the absence of counterions. The N and O signals reflect the counterion content in the doped films, as each anion contains one nitrogen and four oxygen atoms. The weakest N and O signals were observed for the largest $NFSI^{-}$ counterion, consistent with the lowest doping level inferred from the S(2p) XPS spectra.

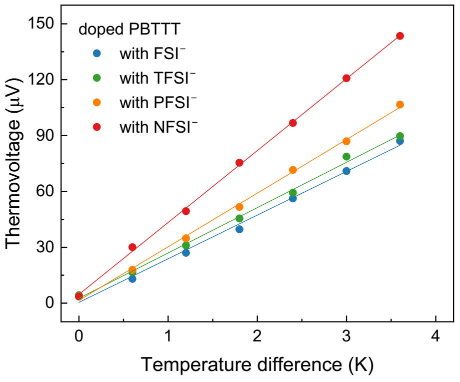

Figure S11. Seebeck coefficients of PBTTT films photocatalytically doped with $Acr\text{-}Me^{+}$ in the presence of various counterions.

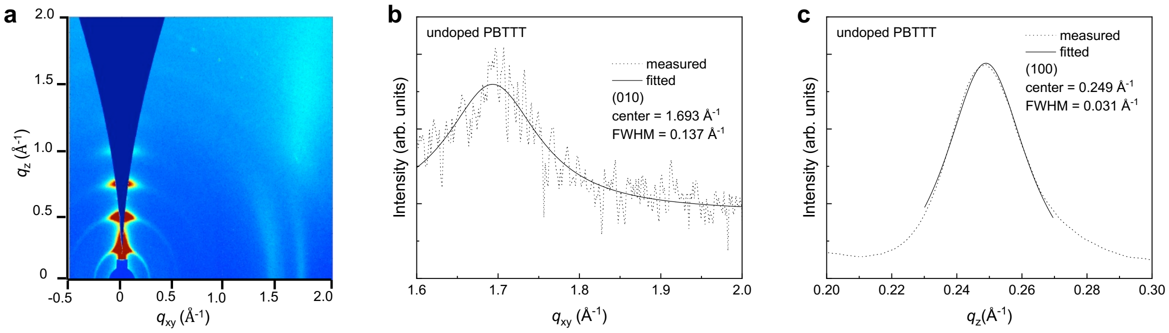

Figure S12. (a) 2D GIWAXS pattern and corresponding (b) π-π stacking and (c) lamellar packing diffraction peak analysis of undoped PBTTT.

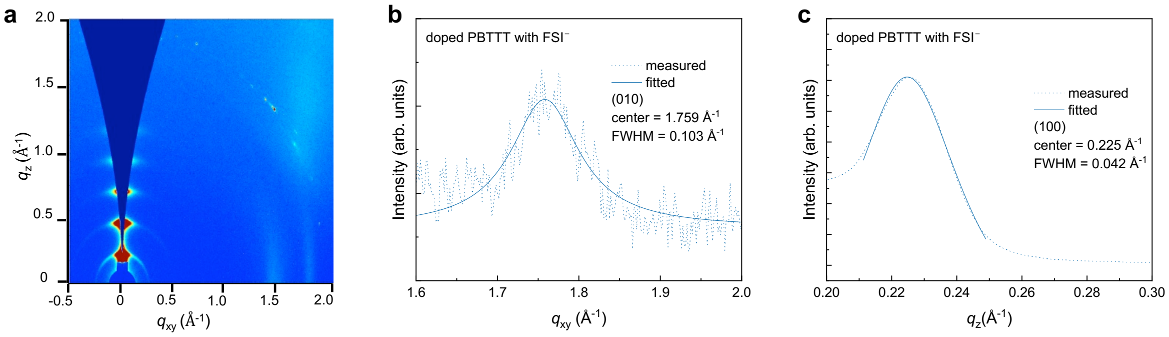

Figure S13. (a) 2D GIWAXS image and corresponding (b) π-π stacking and (c) lamellar packing diffraction peak analysis of PBTTT photocatalytically doped with Acr-Me$^+$ with FSI$^-$.

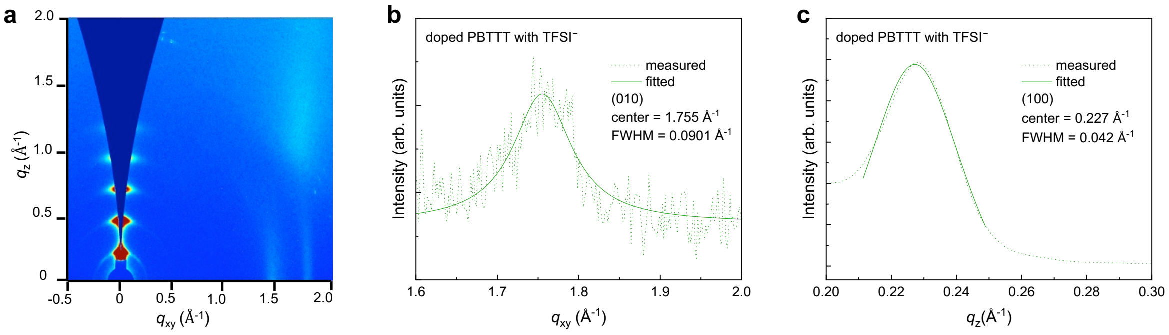

Figure S14. (a) 2D GIWAXS image and corresponding (b) π-π stacking and (c) lamellar packing diffraction peak analysis of PBTTT photocatalytically doped with Acr-Me$^+$ with TFSI$^-$.

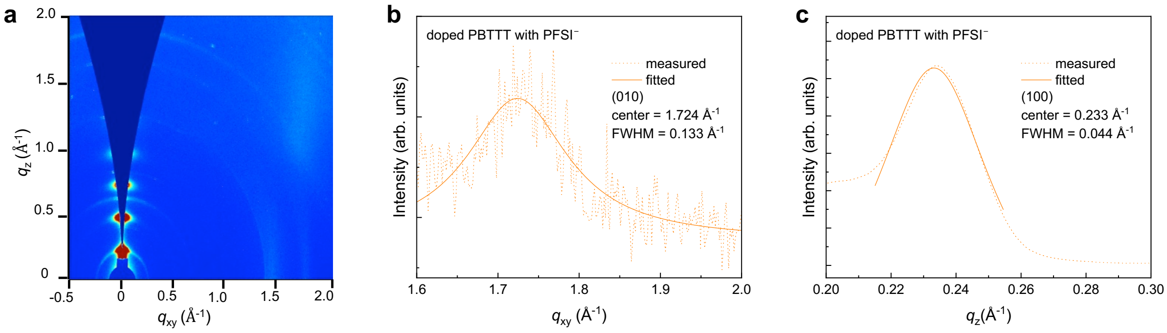

Figure S15. (a) 2D GIWAXS image and corresponding (b) π-π stacking and (c) lamellar packing diffraction peak analysis of PBTTT photocatalytically doped with Acr-Me+ with PFSI–.

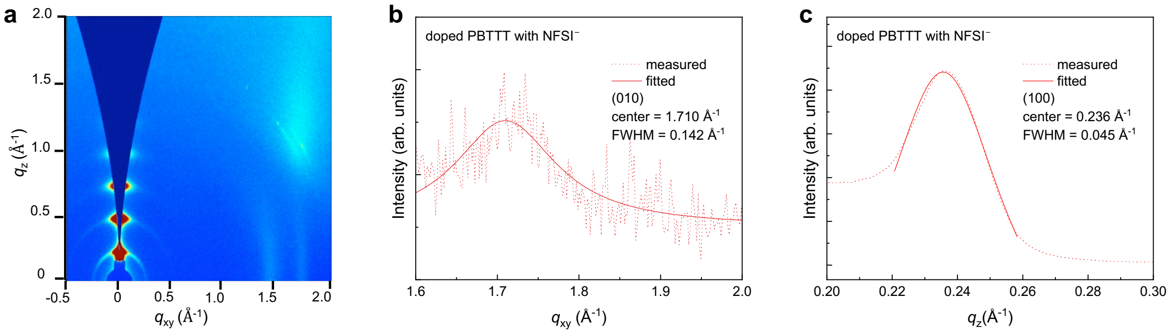

Figure S16. (a) 2D GIWAXS image and corresponding (b) π-π stacking and (c) lamellar packing diffraction peak analysis of PBTTT photocatalytically doped with Acr-Me+ with NFSI–.

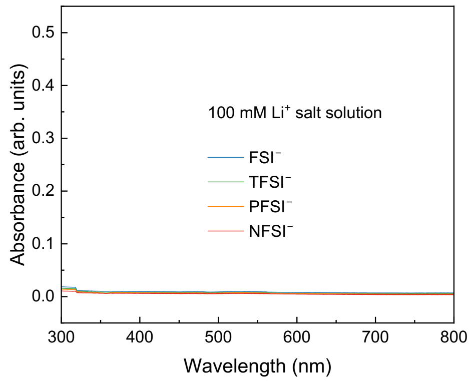

Figure S17. Absorption spectra of Li^+ salt solutions (100 mM) with various counterions.

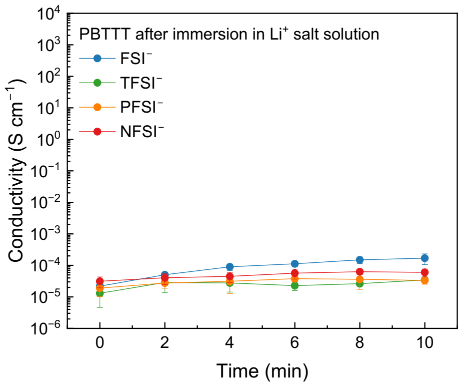

Figure S18. Electrical conductivity of PBTTT films after immersion in a 0.1 M Li+ salt solution in acetonitrile.

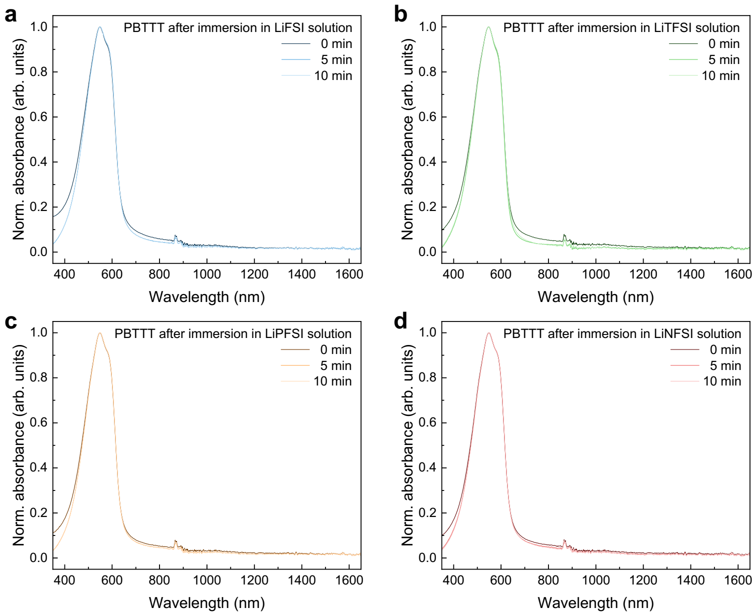

Figure S19. Absorption spectra of PBTTT films after immersion in (a) LiFSI, (b) LiTFSI, (c) LiTFSI, and (d) LiNFSI solutions (0.1 M in acetonitrile).

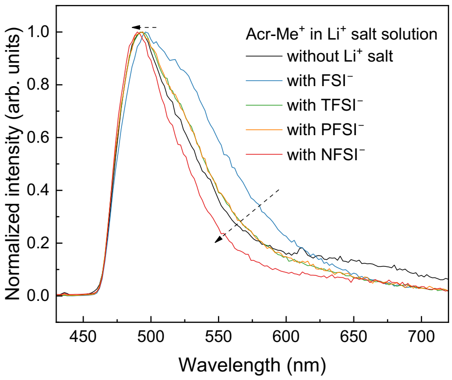

Figure S20. Photoluminescence spectra of Acr-Me$^+$ in Li$^+$ solution with various counterions. The excitation wavelength is 405 nm, and the concentrations of Acr-Me$^+$ and Li$^+$ salt are 10 mM and 100 mM, respectively.

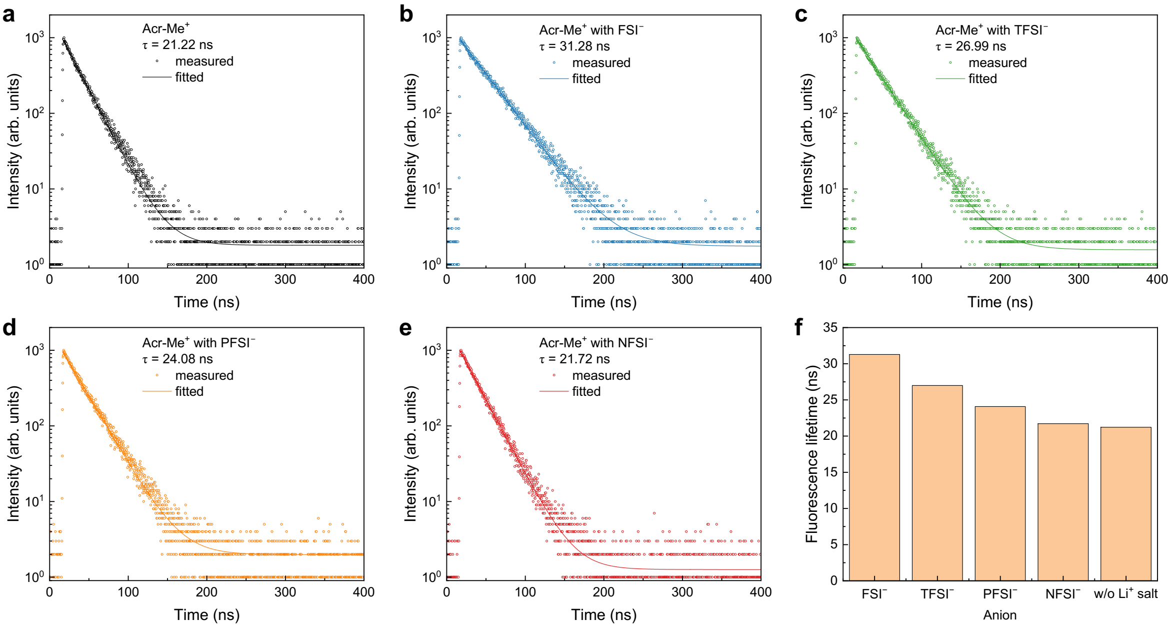

Figure S21. Time-resolved photoluminescence spectra of Acr-Me$^+$ without (a) and with FSI$^-$ (b), TFSI$^-$ (c), PFSI$^-$ (d), and NFSI$^-$ (e). (f) Summary of fluorescence lifetimes of Acr-Me$^+$ in the presence of different counterions. The excitation wavelength is 375 nm, and the concentrations of Acr-Me$^+$ and Li$^+$ salts are 10 mM and 100 mM, respectively.

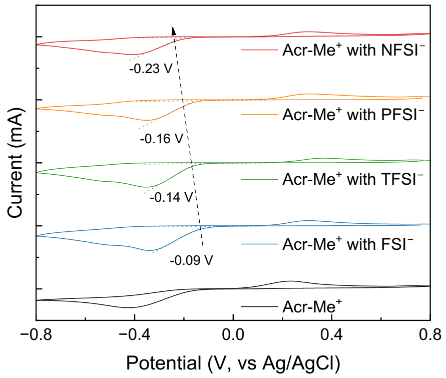

Figure S22. Cyclic voltammograms of Acr-Me$^+$ in Li$^+$ solutions with various counterions (scan rate: 50 mV s^{-1}).

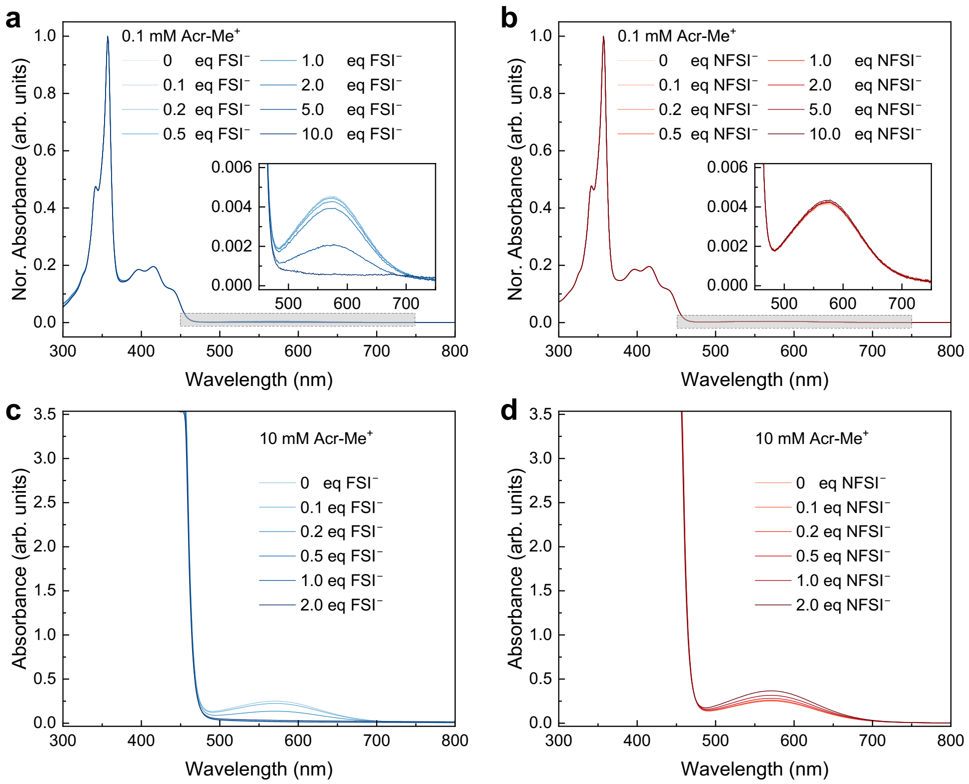

Figure S23. Absorption spectra of diluted (0.1 mM) Acr-Me$^+$ solutions mixed with varying equivalents of FSI$^-$ (a) and NFSI$^-$ (b). Insets show a zoom-in of the 450-750 nm region. Absorption spectra of concentrated (10 mM) Acr-Me$^+$ solutions mixed with varying equivalents of FSI$^-$ (c) and NFSI$^-$ (d).

Table S1. Maximum conductivity of photocatalytically doped *p*-OSC with different counterions.

p-OSC	Photocatalyst	Counterions			
		FSI^- (S cm^{-1})	$TFSI^-$ (S cm^{-1})	$PFSI^-$ (S cm^{-1})	$NFSI^-$ (S cm^{-1})
PBTTT	Acr-Me$^+$	970.62 ± 107.25	659.38 ± 188.86	284.32 ± 79.51	187.82 ± 40.02
PBTTT	Mes-Acr-Me$^+$	858.22 ± 147.06	169.75 ± 30.76	40.91 ± 2.56	7.51 ± 0.26
PBTTT	Mes-Acr-Ph$^+$	807.19 ± 159.29	139.44 ± 20.67	20.72 ± 4.99	6.37 ± 0.93
PgBTTT	Acr-Me$^+$	2005.85 ± 160.36	1049.98 ± 54.96	931.08 ± 32.57	658.38 ± 41.52
P($g_4$2T-T)	Acr-Me$^+$	535.60 ± 20.40	188.43 ± 2.88	157.83 ± 2.71	48.18 ± 3.30

Table S2. Summary of the calculated packing distances, coherence lengths, and paracrystalline disorder parameters for the lamellar and π-π stacking of PBTTT photocatalytically doped by Acr-Me$^+$ with different counterions.

	π-π stacking				Lamellar stacking			
	q_{xy} (010) (Å^{-1})	Distance (Å)	Coherence length (Å)	Paracrystalline disorder	q_z (100) (Å^{-1})	Distance (Å)	Coherence length (Å)	Paracrystalline disorder
Undoped	1.693	3.711	41.276	0.113	0.249	24.135	182.415	0.141
FSI^-	1.759	3.572	54.902	0.097	0.225	27.925	134.640	0.172
$TFSI^-$	1.755	3.580	62.762	0.090	0.227	27.679	134.640	0.172
$PFSI^-$	1.724	3.645	42.518	0.111	0.233	26.966	128.520	0.173
$NFSI^-$	1.710	3.674	39.823	0.115	0.236	26.624	126.664	0.174

Note S1: Density functional theory calculations

Experimentally recorded absorption spectra revealed a new broad band between 450 and 700 nm. This band was already present before adding any Li^+ salts (*i.e.*, in systems containing only ClO_4^-). Upon addition of Li^+ salts, the intensity of this band varied, slightly increasing for $NFSI^-$, decreasing for $TFSI^-$ and $PFSI^-$, and nearly disappearing for FSI^-. To rationalize these findings, we performed DFT simulations of various Acr-Me^+–based systems with different counterions in acetonitrile solvent. We first simulated isolated Acr-Me^+ cations and ion pairs balanced by ClO_4^-, FSI^-, $TFSI^-$, $PFSI^-$, or $NFSI^-$. The optimized geometries and corresponding absorption spectra (Fig. S24) show two dominant absorption peaks at ~350 and ~440 nm, in good agreement with experiment. Some counterions ($TFSI^-$, $PFSI^-$, $NFSI^-$) introduced an additional weak band near 380 nm, but none produced the 450-700 nm feature. Hence, the broad absorption band cannot be attributed to counterion identity alone.

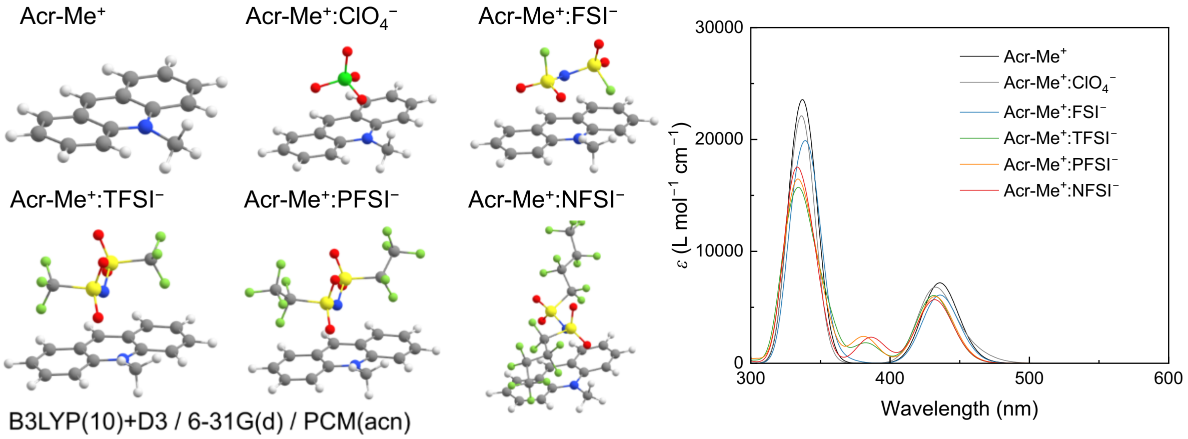

Figure S24. Simulated absorption spectra of Acr-Me^+ with different counterions.

We hypothesized that this feature arises from the aggregation of Acr-Me^+, which is sensitive to the presence of Li^+ salts. To test this, we simulated stacked Acr-Me^+ systems without counterions or salts, considering two arrangements: alternating Me-group orientations (Fig. S25a) and a helix-like stack with Me groups on the same side (Fig. S25b). Both yielded new absorption features above 450 nm, with the helical configuration red-shifted relative to the alternating one. The width of the new band increased with the number of stacked monomers, indicating that Acr-Me^+ aggregates into small crystallites consisting of several units.

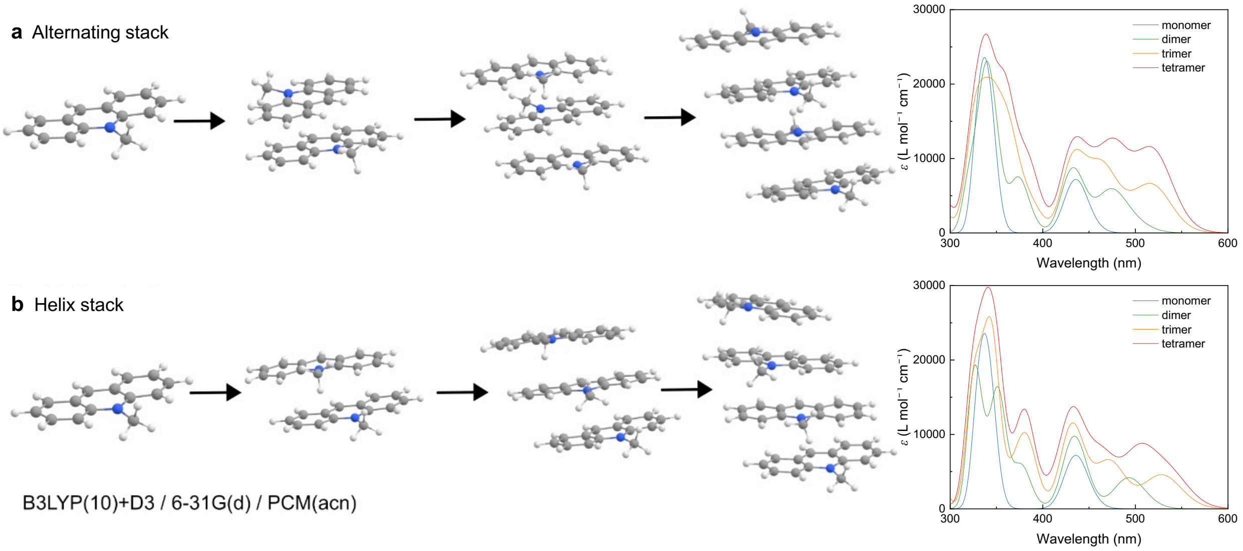

Figure S25. Simulated absorption spectra of stacked Acr-Me$^+$ configurations: (a) alternating and (b) helix-like stacking.

To examine the influence of counterions, we modeled an Acr-Me$^+$ dimer paired with two ClO_4^- ions, which exhibited a new absorption band centered at 470 nm. We then introduced one FSI$^-$ molecule, either coordinating through Li^+ to ClO_4^- above the dimer (Fig. S26a) or positioned laterally (Fig. S26b). While the top-bound geometry increased the intensity of the aggregation band for all salts, lateral placement produced divergent effects: TFSI$^-$, PFSI$^-$, and NFSI$^-$ slightly enhanced the band, whereas FSI$^-$ markedly reduced it, in line with the experimentally observed disappearance of the aggregation-induced feature.

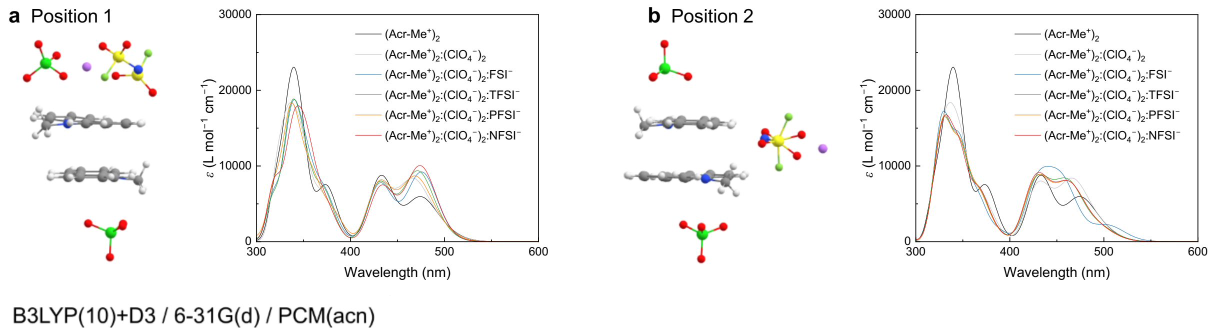

Figure S26. Simulated absorption spectra of an Acr-Me$^+$ dimer with two ClO_4^- counterions and one FSI$^-$ molecule positioned either (a) on top or (b) on the side of the dimer.

Introducing two symmetrically placed salt molecules (Fig. S27) confirmed this trend: the 470 nm band persisted for all salts except FSI$^-$, where it strongly diminished. This suppression is

attributed to the small size of FSI^-, which disrupts π-π stacking by approaching the inter-Acr-Me^+ region. In contrast, the bulkier anions stabilize the dimer via non-covalent interactions. We further analyzed how FSI^- concentration affects stacking geometry. As more FSI^- molecules were introduced, the distance between Acr-Me^+ units increased (Fig. S28a,b), leading to progressive weakening of the aggregation-induced band (Fig. S28c). A manual increase of the inter-molecular distance by 0.3 Å nearly eliminated the band entirely, confirming its extreme sensitivity to π-π stacking.

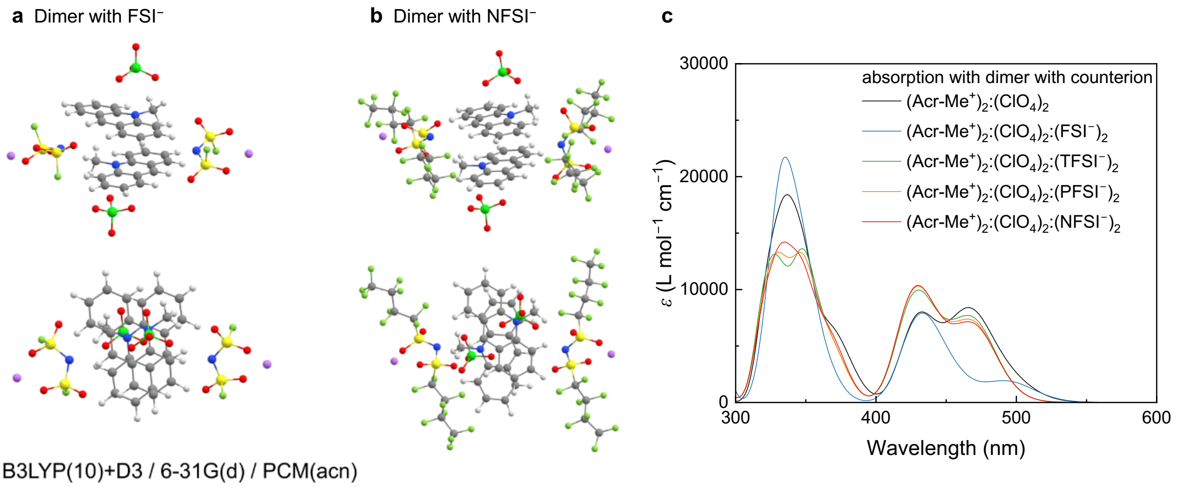

Figure S27. Structures of Acr-Me^+ dimers with two ClO_4^- counterions and two (a) FSI^- or (b) $NFSI^-$ molecules positioned on the sides of the dimer. (c) Corresponding simulated absorption spectra of dimers with two ClO_4^- and two Li^+ salts (FSI^-, $TFSI^-$, $PFSI^-$, or $NFSI^-$) placed laterally.

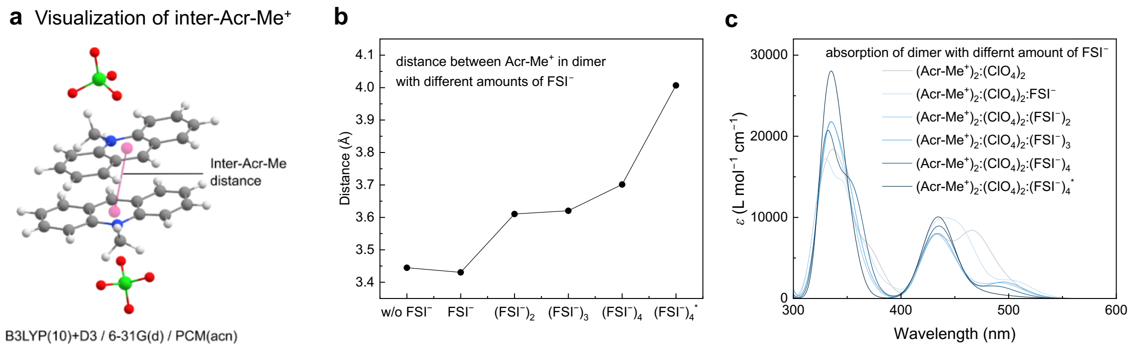

Figure S28. (a) Visualization of how the distance between two Acr-Me^+ molecules was determined. (b) Intermolecular distance in systems containing different numbers of FSI^- molecules. (c) Corresponding absorption spectra of Acr-Me^+ dimers surrounded by varying amounts of FSI^-. All systems were geometrically optimized, except for the one marked with an asterisk (*) in (b) and (c), where the Acr-Me^+ molecules were manually separated by 0.3 Å.

In the $(Acr\text{-}Me^+)_2{:}(ClO_4^-)_2$ system, the lowest-energy aggregation band appeared at 496 nm with an oscillator strength $f = 0.0125$ (HOMO→LUMO transition). Upon adding four FSI^-, the same band shifted slightly to 501 nm, but its intensity dropped significantly ($f = 0.0034$). When the monomers were further separated to 7.7 Å, a weak peak at 506 nm remained, but with negligible oscillator strength. Finally, we estimated the electron affinities of $Acr\text{-}Me^+$ monomers/dimers in the presence of different salts (see Fig. S29 and Table S3). The results revealed a consistent stabilization of the LUMO upon dimer formation.

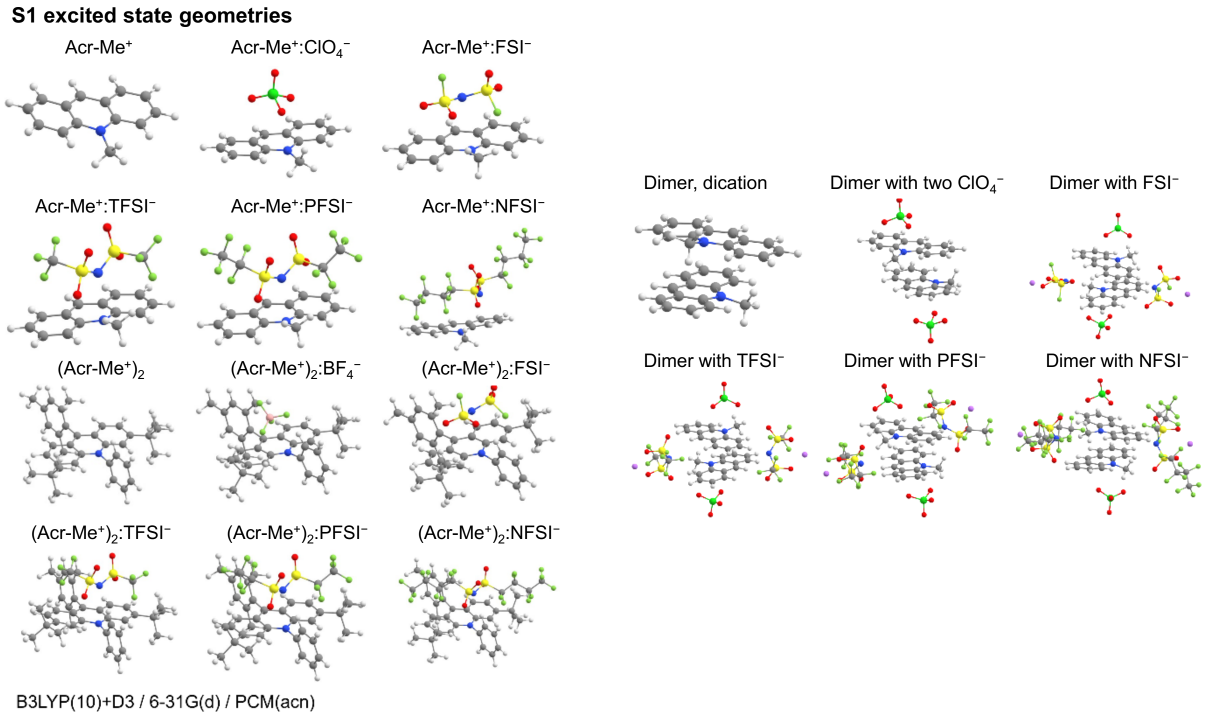

Figure S29. Visualization of S_1-optimized geometries.

In summary, DFT calculations show that the broad absorption band between 450 and 700 nm originates from $Acr\text{-}Me^+$ aggregation. This feature persists for $TFSI^-$, $PFSI^-$, and $NFSI^-$ but disappears upon the addition of FSI^-. The small FSI^- anion disrupts π-π stacking by intruding into the intermolecular region, whereas bulkier anions stabilize the aggregates via non-covalent interactions.

Table S3. LUMO energy values for optimized S_0 and S_1 excited-state geometries.

Compound	LUMO, S_0 [eV]	Dimers	LUMO, S_0 [eV]
$(Acr\text{-}Me^+)_2$	-3.296		
$(Acr\text{-}Me^+)_2:BF_4^-$	-3.235		
$(Acr\text{-}Me^+)_2:FSI^-$	-3.246		
$(Acr\text{-}Me^+)_2:TFSI^-$	-3.206		
$(Acr\text{-}Me^+)_2:PFSI^-$	-3.212		
$(Acr\text{-}Me^+)_2:NFSI^-$	-3.267		
$Acr\text{-}Me^+$	-3.654	$(Acr\text{-}Me^+)_2$	-3.910
$Acr\text{-}Me^+:ClO_4^-$	-3.370	$(Acr\text{-}Me^+)_2:(ClO_4^-)_2$	-3.563
$Acr\text{-}Me^+:FSI^-$	-3.410	$(Acr\text{-}Me^+)_2:(ClO_4^-)_2:(FSI^-)_2$	-3.427
$Acr\text{-}Me^+:TFSI^-$	-3.329	$(Acr\text{-}Me^+)_2:(ClO_4^-)_2:(TFSI^-)_2$	-3.360
$Acr\text{-}Me^+:PFSI^-$	-3.335	$(Acr\text{-}Me^+)_2:(ClO_4^-)_2:(PFSI^-)_2$	-3.355
$Acr\text{-}Me^+:NFSI^-$	-3.321	$(Acr\text{-}Me^+)_2:(ClO_4^-)_2:(NFSI^-)_2$	-3.357

Compound	LUMO, S_1 [eV]	Dimers	LUMO, S_1 [eV]
$(Acr\text{-}Me^+)_2$	-3.671		
$(Acr\text{-}Me^+)_2:BF_4^-$	-3.424		
$(Acr\text{-}Me^+)_2:FSI^-$	-3.437		
$(Acr\text{-}Me^+)_2:TFSI^-$	-3.392		
$(Acr\text{-}Me^+)_2:PFSI^-$	-3.400		
$(Acr\text{-}Me^+)_2:NFSI^-$	-3.450		
$Acr\text{-}Me^+$	-3.874	$(Acr\text{-}Me^+)_2$	-4.213
$Acr\text{-}Me^+:ClO_4^-$	-3.621	$(Acr\text{-}Me^+)_2:(ClO_4^-)_2$	-3.829
$Acr\text{-}Me^+:FSI^-$	-3.613	$(Acr\text{-}Me^+)_2:(ClO_4^-)_2:(FSI^-)_2$	-3.678
$Acr\text{-}Me^+:TFSI^-$	-3.550	$(Acr\text{-}Me^+)_2:(ClO_4^-)_2:(TFSI^-)_2$	-3.688
$Acr\text{-}Me^+:PFSI^-$	-3.554	$(Acr\text{-}Me^+)_2:(ClO_4^-)_2:(PFSI^-)_2$	-3.641
$Acr\text{-}Me^+:NFSI^-$	-3.560	$(Acr\text{-}Me^+)_2:(ClO_4^-)_2:(NFSI^-)_2$	-3.686
